# Impact of Obstructive Sleep Apnea on In-Stent Restenosis in Coronary Heart Disease Patients after Elective Drug-Eluting Stenting

**DOI:** 10.31083/RCM25814

**Published:** 2025-01-15

**Authors:** Wenjie Yang, Zhuoshan Huang, Ke Yang, Dinghui Liu, Junpeng Xiao, Zhen Wu, Ling Jiang, Shan Cao, Xujing Xie, Shujie Yu

**Affiliations:** ^1^Department of Cardiology, The Third Affiliated Hospital of Sun Yat-sen University, 510630 Guangzhou, Guangdong, China

**Keywords:** in-stent restenosis, obstructive sleep apnea, drug-eluting stent

## Abstract

**Background:**

Extensive research has established obstructive sleep apnea (OSA) as a contributing factor to numerous cardiovascular and cerebrovascular diseases. However, whether OSA affects in-stent restenosis (ISR) after elective drug-eluting stenting is unclear. Therefore, the objective of this study was to examine the impact of OSA on ISR in patients with coronary heart disease (CHD) who underwent successful elective drug-eluting stent (DES) implantation.

**Methods:**

This study retrospectively analyzed CHD patients who successfully underwent elective coronary stent implantation and overnight sleep breathing monitoring and were readmitted for coronary angiography due to symptoms of CHD at 12 to 26 months after percutaneous coronary intervention (PCI). OSA was diagnosed when the apnea-hypopnea index (AHI) was ≥5 events/hour. ISR was defined as >50% restenosis of the vessel diameter in which the DES was implanted. To explore the association between OSA and ISR among patients with CHD, multivariate logistic regression models were developed and utilized.

**Results:**

This study enrolled 206 individuals who were diagnosed with CHD, with a mean age of 62.01 ± 10.27 years, and males constituted 76.2% of the patient population. After a median follow-up period of 15 months following DES implantation, there was a significant increase in the incidence of ISR among patients with moderate to severe OSA, increasing from 10.9% to 31.3% (*p* < 0.001). According to the fully adjusted model, the occurrence of ISR was found to be independently associated with the presence of OSA (OR: 3.247, 95% CI: 1.373–7.677, *p* = 0.007).

**Conclusions:**

In individuals who underwent elective drug-eluting stenting, OSA is an independent risk factor for ISR.

## 1. Introduction

From the first case of balloon angioplasty to the introduction of the latest generation of drug-eluting stents (DESs), the evolution of percutaneous coronary intervention (PCI) alongside coronary stents has undergone substantial innovation and refinement [1]. The invention of new-generation DESs has significantly improved PCI outcomes, markedly reducing post-PCI mortality, in-stent restenosis (ISR) incidence, the need for prolonged dual antiplatelet therapy, and ischemic complications, thereby greatly enhancing the efficacy of PCI [2, 3]. However, despite significant improvements in the antirestenotic performance of DESs, as well as technological innovations in PCI, medication, and other secondary prevention strategies, ISR remains a major challenge after PCI, with approximately 2%–20% of patients experiencing ISR within five years of the procedure, especially when stents are implanted in anatomically complex coronary arteries [4, 5, 6, 7, 8]. Therefore, identifying ISR risk factors and implementing targeted preventive strategies are highly important.

Obstructive sleep apnea (OSA) is a prevalent sleep-related breathing disorder. It is typified by repeated incidents of both complete and partial blockages of the upper airway, which in turn cause periodic drops in blood oxygen levels, fluctuations in autonomic nervous system activity, and disruptions in sleep continuity [9, 10]. There is accumulating evidence that OSA is correlated with many cardiovascular complications, including pulmonary hypertension, hypertension, coronary heart disease (CHD), stroke, atrial fibrillation and other arrhythmias, heart failure, and cardiovascular death, due to physiological changes such as hypoxia, hypercapnia, and arousal [11, 12, 13, 14]. In patients diagnosed with cardiovascular conditions including arrhythmia, heart failure, CHD, and hypertension, the occurrence rate of OSA ranges from 50% to 83% [12]. Individuals with OSA are at an elevated risk of adverse outcomes following PCI. Specifically, there is a significantly greater frequency of major adverse cardiac events (MACEs) among those with OSA [15, 16, 17]. The severity of OSA is also correlated with patient prognosis after PCI. Patients suffering from acute coronary syndrome (ACS) who are diagnosed with moderate to severe OSA exhibit a poorer prognosis than those with no or mild OSA [18]. Research indicates that during a 6-month follow-up period of ACS patients who underwent implantation of bare-metal stents, those with OSA displayed a significantly greater degree of late loss and a greater rate of binary restenosis in quantitative coronary angiography assessments than did their counterparts without OSA [19].

To our knowledge, the relationship between OSA and ISR after DES implantation has yet to be clearly defined. There appears to be a lack of targeted research specifically aimed at investigating this association. Therefore, the objective of this study was to examine the impact of OSA on ISR in patients with CHD who underwent successful elective DES implantation. We hypothesized that OSA is an independent risk factor for ISR. The incidence of ISR is significantly higher in patients with OSA than in those without OSA. This study is novel in the field as it is the first to systematically assess the independent influence of OSA in the development of ISR. Furthermore, understanding this association has important clinical implications, as it may influence screening strategies and management options for patients at high risk for cardiovascular events, possibly leading to early intervention to reduce the risk of ISR.

## 2. Materials and Methods

### 2.1. Study Population

CHD patients who underwent successful elective drug-eluting stenting and overnight sleep respiratory monitoring from January 2017 to December 2022 at the Third Affiliated Hospital of Sun Yat-Sen University and who were readmitted for follow‑up coronary angiography due to symptoms of CHD ranging from 12 to 26 months after successful stent implantation were retrospectively reviewed.

We excluded patients who met any of the following criteria: (1) aged less than 18 years; (2) had malignant tumors, mental illness, or chronic pain; (3) received continuous positive airway pressure (CPAP) treatment; (4) had a history of PCI or coronary artery bypass grafting; (5) had culprit lesions treated with bioabsorbable scaffolds or bare-metal stents; (6) had stent thrombosis and stent rupture after PCI; (7) had undergone PCI for calcified or bifurcation lesions; (8) had an estimated glomerular filtration rate (eGFR) <30 mL/min/1.73 m^2^, active liver disease, or severe infection; and (9) had dementia or cognitive dysfunction. Patients without sufficient clinical data were excluded from this study. Most importantly, this retrospective study was conducted in accordance with the Declaration of Helsinki. The Institutional Review Board (IRB) of our hospital, granted approval for this study (Ethics Approval Number: [2022]02-121-01). In addition, written/oral informed consent was also obtained from all participants.

### 2.2 Coronary Angiography and Intervention

The study ensured that each participant was administered appropriate preoperative doses of aspirin and clopidogrel. This included taking aspirin at a dose of 0.1 g daily for a minimum of three days or receiving a loading dose of 0.3 g, along with clopidogrel at a dose of 75 mg daily for at least four days or a loading dose of 300 mg. Initial evaluations of patients were carried out through coronary angiography, after which the necessity for PCI was determined. This decision was made by two experienced interventional cardiologists in accordance with the guidelines set forth in the Chinese manual for PCI [20]. Before the procedure commenced, patients received an initial dose of 100 U/kg unfractionated heparin (UFH), with an additional infusion of 1000 U/hour provided if the procedure extended beyond one hour. The choice regarding the type and dimensions of the stent to be implanted was left to the discretion of the performing operators. Details regarding the PCI, including the quantity of vessels undergoing intervention, the number of stents used, the cumulative length of all stents, and the pressure applied during postprocedural balloon dilation, were meticulously documented. After the intervention, the implementation of secondary prevention measures, as recommended by current guidelines, was guaranteed.

### 2.3 Sleep Study and OSA

All patients included in the analysis underwent respiratory sleep monitoring overnight in the ward using a portable sleep monitoring device (RS01, CONTEC, Qinhuangdao, China) due to clinical suspicion of OSA when their clinical condition was stable after selective DES implantation. The device captured information on nasal airflow, arterial oxygen saturation, and occurrences of snoring. The monitoring data were then analyzed independently by a trained physician who was unaware of the clinical characteristics of the patients to ensure an unbiased assessment and to generate an analysis report. Sleep apnea was characterized by a total halt in nasal airflow for 10 seconds or more, while hypopnea was identified as a decrease in nasal airflow by at least 50%, but not completely stopping, for a duration of at least 10 seconds [9]. This reduction in airflow during hypopnea was also associated with a decrease in blood oxygen saturation of at least 4%. The apnea-hypopnea index (AHI) was determined by calculating the sum of apnea and hypopnea episodes that occur per hour of sleep. To ensure consistency in sleep monitoring, all patients were advised to adhere to their regular sleeping patterns and habits. OSA was diagnosed when the AHI reached or exceeded 5 events per hour [10]. OSA was classified into different severity levels: mild OSA was indicated by an AHI ranging from 5 to 15 events/hour, moderate OSA was denoted by an AHI falling between 15 and 30 events/hour, and severe OSA was characterized by an AHI that exceeds 30 events/hour [21]. Furthermore, evidence from prior research has demonstrated that individuals diagnosed with moderate to severe OSA tend to have a poorer prognosis than those with no or mild OSA [18]. Therefore, we grouped patients into either moderate and severe OSA patients (moderate-severe group, AHI ≥15 events/hour) or normal and mild OSA patients (normal-mild group, AHI <15 events/hour) [22, 23].

### 2.4 Follow‑Up Angiography and Evaluation of ISR and Non-ISR

All patients included in this analysis were subjected to a follow-up coronary angiography due to symptoms such as chest pain, chest tightness, or other manifestations related to CHD. This follow-up occurred within a timeframe extending from 12 to 26 months after the implantation of DESs. The assessment of ISR necessitates examination through coronary angiography. Interpretation of the angiographic findings obtained during follow-up, as well as identification of ISR, was conducted by two experienced coronary intervention cardiologists. Any discrepancies that arose during the identification of ISR were resolved through consultation with a third cardiologist. ISR was considered the occurrence of significant diameter stenosis (>50%) within the stent segment, in accordance with prior research [6].

### 2.5 Data and Definitions

We collected demographic and clinical data, including sex, age, smoking and drinking status, body mass index (BMI), left ventricular ejection fraction (LVEF), previous medical history, medications at discharge, coronary angiography and stent implantation procedure parameters, and sleep information, for all enrolled patients from our electronic health care system. All enrolled patients underwent venous blood sampling after a fast of more than 8 hours after admission. We collected information, including eGFR, uric acid (UA), and glycosylated hemoglobin A1c (HbA1c), as well as blood lipid information on patient blood samples analyzed by the biochemical laboratory of our hospital.

Individuals were classified as having diabetes mellitus based on a prior diagnosis of the condition, current use of glucose-lowering treatments, or the presence of classic symptoms alongside a fasting blood glucose level exceeding 7.1 mmol/L and/or a random blood glucose level surpassing 11.1 mmol/L [24]. Hypertension was identified either through a historical diagnosis or by measuring blood pressure at 140/90 mmHg or above on three separate occasions [25]. BMI was calculated using the formula weight in kilograms divided by height in meters squared (kg/m^2^). A BMI of 28 kg/m^2^ or higher was considered to indicate obesity. The term “multiple stents” refered to the use of two or more DESs in a patient. Multivessel disease manifested as significant stenosis—defined as a 50% or greater reduction in diameter—impacting the left main (LM) coronary artery or involving at least two of the following: the right coronary artery (RCA), left anterior descending (LAD), or left circumflex (LCX), as determined by coronary angiography. The coronary artery in which the stent was implanted was defined as the interventional vessel.

### 2.6 Statistical Analysis 

We used the mean ± standard deviation (SD) for reporting continuous variables with a normal distribution. For non-normally distributed variables, medians [interquartile ranges (IQRs)] were reported. The student *t* test was used to compare means of continuous variables, and the Wilcoxon Mann-Whitney rank sum test was used to compare the medians of nonparametric data. Absolute numbers (percentages) were used for categorical variables. For categorical data, differences between groups were evaluated using either the Fisher’s exact test or the chi-squared test.

We first performed univariate logistic regression analysis to evaluate the associations between ISR and baseline variables. Any baseline variable that demonstrated a *p* value less than 0.05 in the univariate logistic regression analysis or was deemed clinically significant for ISR was included in the multivariate logistic regression analysis. Logistic models were constructed to control for confounders and explore the relationship between ISR and OSA (analyzed as a continuous and categorical variable).

All analyses were carried out using SPSS 25.0 (IBM, Armonk, NY, USA). A value of *p* < 0.05 was considered statistically significant.

## 3. Results

### 3.1 Baseline Characteristics and Sleep Information

This study ultimately included 206 patients. Table 1 displayed their baseline characteristics along with the procedural parameters. The mean age of the participants was 62.01 ± 10.27 years. There were 157 male patients (76.2%). The prevalence of diabetes and hypertension was 53.9% and 63.1%, respectively. The average BMI was 24.58 ± 3.16 kg/m^2^, and 21 patients were obese, accounting for 10.2% of the total study population. The utilization rate of statins and aspirin was close to 100%. In addition, 77.2% of the patients had more than two coronary main branch diseases, and 20 patients had LM disease. The median minimal stent diameter was 2.75 mm, and the median total stent length was 48 mm. A total of 56.3% of patients underwent angiographic implantation of more than two stents, while 10.7% had an overlap between stents.

**Table 1.** **Baseline information for patients grouped by AHI**.

Variables		All (n = 206)	AHI <15 (n = 110)	AHI ≥15 (n = 96)	*p *value
Age, year		62.01 ± 10.27	61.85 ± 10.39	62.01 ± 10.18	0.801
Male, n (%)		157 (76.2)	79 (71.8)	78 (81.3)	0.113
BMI, kg/m^2^		24.58 ± 3.16	23.60 ± 2.28	25.70 ± 3.40	<0.001
Obesity, n (%)		21 (10.2)	2 (1.8)	19 (19.8)	<0.001
Hypertension, n (%)		130 (63.1)	65 (59.1)	65 (67.7)	0.201
Diabetes mellitus, n (%)		111 (53.9)	58 (52.7)	53 (55.2)	0.722
Current smoking, n (%)		62 (30.1)	34 (30.9)	28 (29.2)	0.768
Current drinking, n (%)		36 (17.5)	20 (18.2)	16 (16.7)	0.775
TC, mmol/L		4.40 [3.52, 5.42]	4.18 [3.46, 5.51]	4.62 [3.59, 5.40]	0.315
TG, mmol/L		1.51 [1.12, 2.35]	1.44 [1.07, 2.09]	1.73 [1.24, 2.44]	0.040
HDL-C, mmol/L		0.94 ± 0.24	0.96 ± 0.25	0.92 ± 0.22	0.311
LDL-C, mmol/L		2.88 ± 1.10	2.81 ± 1.20	2.96 ± 0.97	0.337
LP (a), mg/L		151 [87, 312]	141 [84, 311]	158 [94, 334]	0.357
eGFR, mL/min/1.73 m^2^		87.8 [72.5, 95.4]	88.5 [76.5, 96.7]	84.1 [68.0, 92.2]	0.041
UA, umol/L		397.4 ± 114.2	402.2 ± 128.2	391.8 ± 96.1	0.519
HbA1c		6.1 [5.6, 6.8]	6.1 [5.6, 6.7]	5.9 [5.5, 7.1]	0.787
Hemoglobin, g/L		136 [127, 146]	135 [129, 145]	138 [126, 146]	0.795
LVEF		66 [60, 69]	66 [61, 69]	65 [59, 70]	0.330
Medications at discharge					
	Aspirin, n (%)	183 (98.5)	108 (98.2)	95 (99.0)	1.000
	Ticagrelor, n (%)	68 (33.0)	41 (37.3)	27 (28.1)	0.164
	Clopidogrel, n (%)	137 (66.5)	69 (62.7)	68 (70.8)	0.219
	Statin, n (%)	204 (99.0)	110 (100)	94 (97.9)	0.216
	β-block, n (%)	127 (61.7)	63 (57.3)	64 (66.7)	0.167
	ACEI/ARB, n (%)	99 (48.1)	54 (49.1)	45 (46.9)	0.751
Angiography					
	Multivessel disease, n (%)	159 (77.2)	85 (77.3)	74 (77.1)	0.974
	LM disease, n (%)	20 (9.7)	12 (10.9)	8 (8.3)	0.533
Intervention vessel					
	LM, n (%)	8 (3.9)	5 (4.5)	3 (3.1)	0.869
	LAD, n (%)	118 (57.3)	64 (58.2)	54 (56.3)	0.780
	LCX, n (%)	66 (32.0)	37 (33.6)	29 (30.2)	0.599
	RCA, n (%)	82 (39.8)	44 (40.0)	38 (39.6)	0.951
Type of drug-eluting stent					
	Sirolimus stent, n (%)	157 (76.2)	87 (79.1)	70 (72.9)	0.299
	Everolimus stent, n (%)	50 (24.3)	24 (21.8)	26 (27.1)	0.379
	Multiple stents (n ≥2), n (%)	116 (56.3)	61 (55.5)	55 (57.3)	0.791
	Minimal stent diameter, mm	2.75 [2.50, 3.00]	2.75 [2.50, 3.00]	2.75 [2.50, 3.00]	0.595
	Overlapping stents, patients (%)	22 (10.7)	9 (8.2)	13 (13.5)	0.214
	Maximal expansion pressure	16 [16, 18]	16 [16, 18]	18 [16, 18]	0.313
	Total stent length, mm/patients	48 [30, 69]	49 [29, 70]	47 [30, 65]	0.727
	ISR, n (%)	42 (20.4)	12 (10.9)	30 (31.3)	<0.001

AHI, apnea-hypopnea index; BMI, body mass index; TC, total cholesterol; TG, triglyceride; HDL-C, high-density lipoprotein cholesterol; LDL-C, low-density lipoprotein cholesterol; LP (a), lipoprotein a; eGFR, estimated glomerular filtration rate; UA, uric acid; HbA1c, hemoglobin A1c; LVEF, left ventricular ejection fraction; ACEI/ARB, angiotensin-converting enzyme inhibitors/angiotensin receptor blocker; LM, left main artery; LAD, left anterior descending artery; LCX, left circumflex artery; RCA, right coronary artery; ISR, in-stent restenosis.

Table 1 illustrates the division of the enrolled patients into two groups. Individuals with moderate-severe OSA had a greater BMI than those with normal or mild OSA. Obesity was also observed in a greater percentage of patients diagnosed with moderate-severe OSA. Additionally, notable differences in triglyceride (TG) levels and eGFR were detected among the groups. Furthermore, the correlation between the incidence of ISR and the AHI was considerably greater (10.9% vs. 31.3%, *p* < 0.001). Table 2 showed the sleep information for all patients. Patients with moderate-severe OSA had a greater AHI and lower minimal SaO_2_ during sleep than those with normal-mild OSA, and the total duration of SaO_2_ <90% was longer during sleep (*p* < 0.001). There was a significant difference in the mean SaO_2_ (*p* = 0.029).

**Table 2.** **Sleep information**.

Variables	All (n = 206)	AHI <15 (n = 110)	AHI ≥15 (n = 96)	*p *value
AHI, events/h	12.60 [6.89, 21.51]	7.31 [3.85, 10.54]	22.05 [17.66, 31.67]	<0.001
Minimal SaO_2_ (%)	85.0 [82.0, 89.0]	87.0 [85.0, 90.0]	83.0 [80.0, 86.0]	<0.001
Mean SaO_2_ (%)	94.0 [93.0, 95.0]	94.5 [93.0, 96.0]	94.0 [93.0, 95.0]	0.029
Total percentage of time of SaO_2_ <90%	0.44 [0.02, 2.60]	0.13 [0.00, 0.66]	1.63 [0.29, 5.81]	<0.001

AHI, apnea-hypopnea index.

Additionally, we analyzed the differences between the ISR and non-ISR groups (**Supplementary Table 1**). Compared to non-ISR patients, those with ISR were more likely to have two or more stents implanted, a longer total length of the implanted stent, and greater pressure of stent expansion after implantation. The incidence of overlap between stents was significantly greater in ISR patients (26.2% vs. 6.7%, *p* < 0.001).

### 3.2 The Incidence of ISR after Elective Coronary Drug-Eluting Stenting and Comparison of Sleep Parameters between the ISR and Non-ISR Groups

**Fig. 1.**
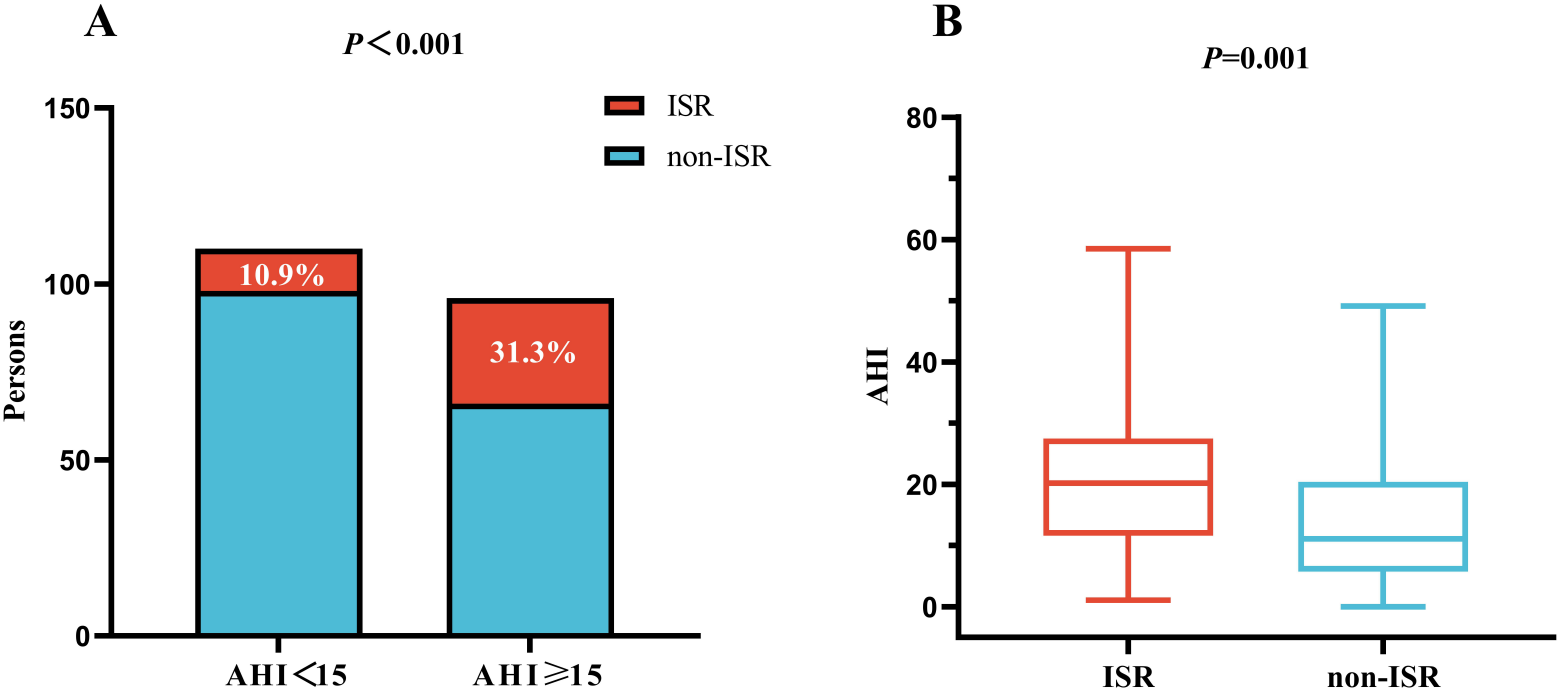
**Impact of the AHI on the incidence of DES-ISR (A) and a comparison of the AHI between the ISR and non-ISR groups (B)**. AHI, apnea-hypopnea index; ISR, in-stent restenosis; DES, drug-eluting stent.

**Fig. 2.**
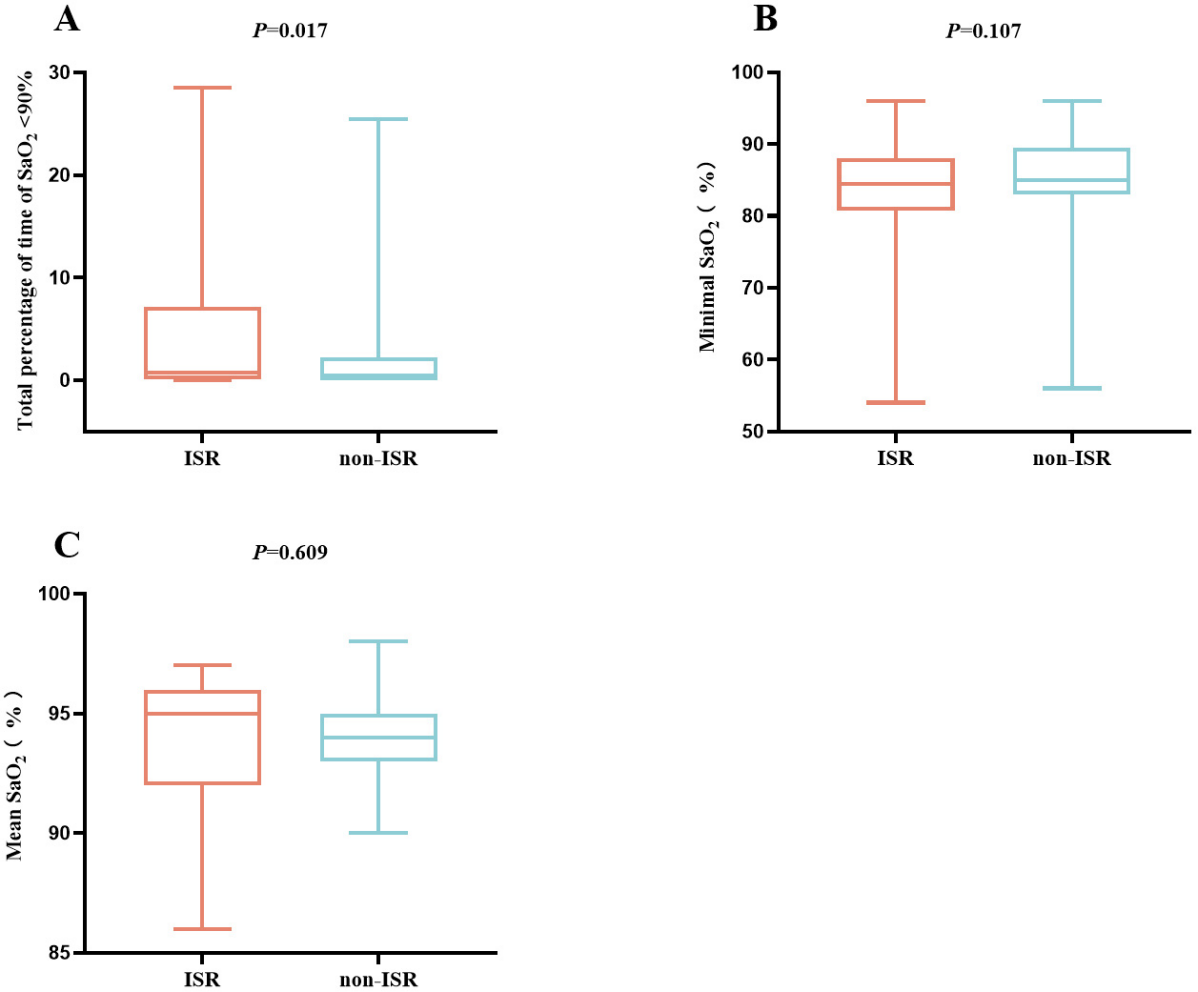
**The comparison of the total percentage of time of SaO_2_ <90% (A), minimal SaO_2_ (B), and mean SaO_2_ (C) level between the ISR and non-ISR groups in the overall study population**. ISR, in-stent restenosis.

As shown in Fig. 1A, the incidence of ISR increased with increasing AHI (10.9% vs. 31.3%, *p* < 0.001). Additionally, the AHI was significantly greater in the group with ISR than in the group without ISR (*p* = 0.001, Fig. 1B). We also found that patients with ISR had a longer duration for which SaO_2_ was <90% of that during the total sleep time (*p* = 0.017), and the percentage of time with SaO_2_ <90% in non-ISR patients was closer to 0% (Fig. 2A). In addition, patients with ISR had a lower median minimal SaO_2_ (94% vs. 95%, *p* = 0.107) during overnight sleep (Fig. 2B), although the difference was not statistically significant (Fig. 2C).

**Fig. 3.**
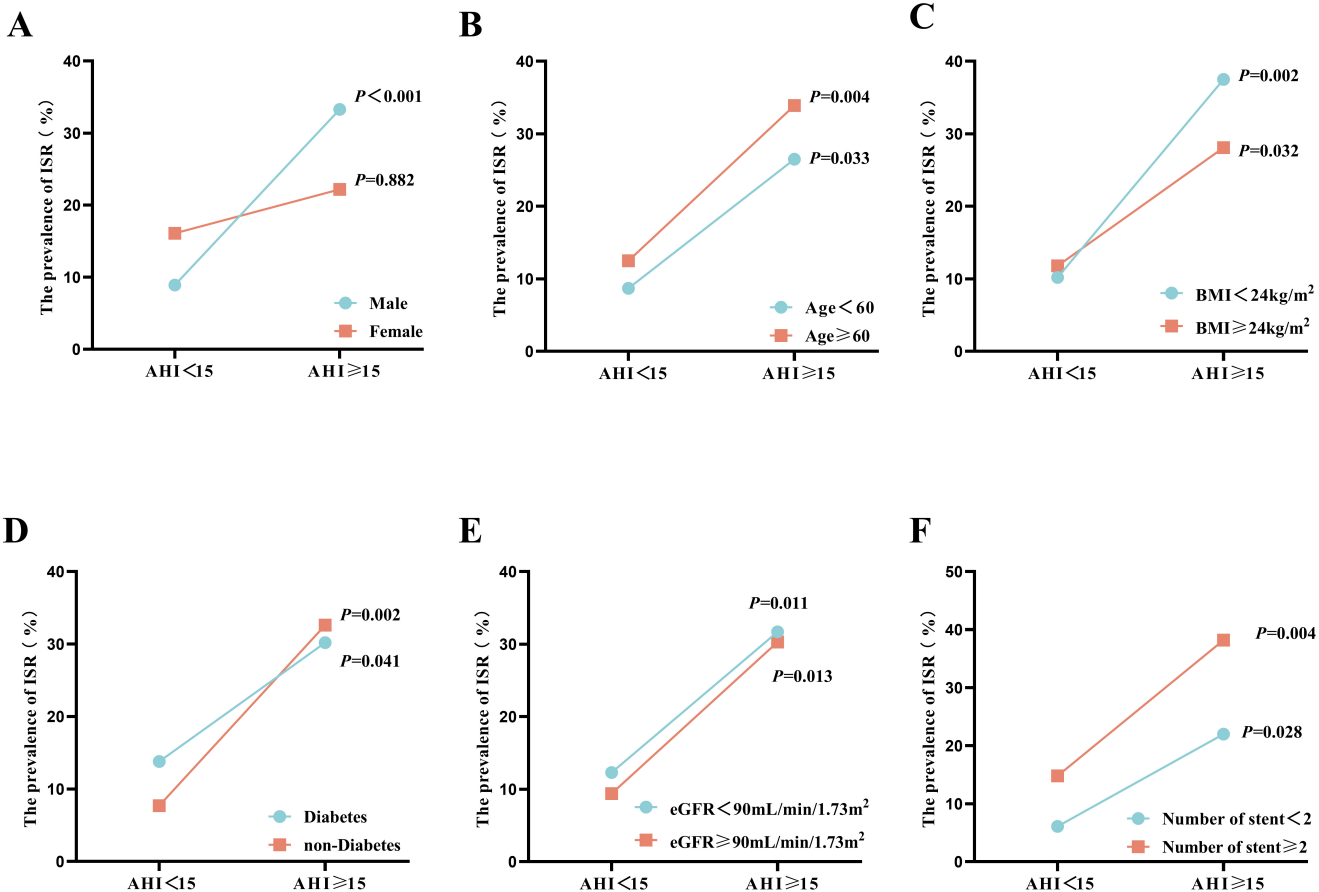
**Impact of OSA on the incidence of ISR in subgroups stratified by sex (A), age (B), BMI (C), diabetes status (D), eGFR (E), and number of stents (F)**. ISR, in-stent restenosis; AHI, apnea-hypopnea index; BMI, body mass index; eGFR, estimated glomerular filtration rate; OSA, obstructive sleep apnea.

The impact of OSA on the incidence of ISR was also analyzed in different subgroups. As shown in Fig. 3, subgroup analysis revealed that male sex (8.9% vs. 33.3%, *p* < 0.001), age ≥60 years (12.5% vs. 33.9%, *p* = 0.004), BMI <24 kg/m^2^ (10.2% vs. 37.5%, *p = *0.002), the presence of diabetes (13.8% vs. 30.2%, *p* = 0.036), an eGFR <90 mL/min/1.73 m^2^ (12.3% vs. 31.7%, *p* = 0.011), and the presence of ≥2 stents (14.8% vs. 38.2%, *p* = 0.004) were correlated with ISR. In the subgroups of patients aged <60 years with a BMI ≥24 kg/m^2^, a nondiabetes status, an eGFR ≥90 mL/min/1.73 m^2^, and <2 stents were included.

### 3.3 Associations between OSA and ISR According to Univariate Analysis

The univariate logistic regression analysis incorporated both clinically relevant variables and sleep parameters that showed potential importance (**Supplementary Table 2**). Our study revealed an association between the AHI and the risk of ISR after PCI, with the risk increasing by 1 unit for every 1 unit increase in the AHI (OR: 1.044, 95% CI: 1.017–1.071, *p* = 0.001). **Supplementary Fig. 1** presented the analysis of the receiver operating characteristic (ROC) curve, indicating that the AHI possesses a moderate ability to predict DES-ISR in patients with CHD who have undergone PCI. This was evidenced by the area under curve (AUC) of 0.662 (95% CI: 0.569–0.756, *p* = 0.001).

By categorizing the AHI and employing an AHI value of less than 15 (representing no to mild OSA) as the reference, it was observed that the occurrence of DES-ISR was elevated in patients with an AHI of 15 or above, which was indicative of moderate to severe OSA (OR: 3.712, 95% CI: 1.774–7.770, *p* = 0.001). Moreover, there was a significant association between DES-ISR and the presence of overlapping stents (OR: 4.935, 95% CI: 1.966–12.392, *p* = 0.001). Correlations were also found for the number of stents, total stent length, multiple stents, and total percentage of time of SaO_2_ <90% (**Supplementary Table 2**).

### 3.4 Associations between OSA and ISR According to Multivariate Analysis

Multivariate logistic regression models were constructed to examine the AHI initially as a continuous variable in this research. Table 3 revealed noteworthy distinctions between the outcomes of Model 1 (OR: 1.044, 95% CI: 1.015–1.074, *p* = 0.002) and Model 2 (OR: 1.036, 95% CI: 1.003–1.069, *p = *0.032). The analysis showed that each unit increase in the AHI is associated with an increased risk of ISR. Even in the fully adjusted model (Model 3), the association between the AHI and ISR remained significant, with the risk of ISR increasing by approximately one-fold for every one-unit increase in the AHI (OR: 1.035, 95% CI: 1.002–1.068, *p* = 0.037).

**Table 3.** **Association of AHI or OSA with ISR in multivariate logistic regression**.

		OR	95% CI	*p* value
Model 1				
	AHI, per 1-unit increase	1.044	1.015–1.074	0.002
	OSA	3.751	1.706–8.251	0.001
Model 2				
	AHI, per 1-unit increase	1.036	1.003–1.069	0.032
	OSA	3.253	1.385–7.642	0.007
Model 3				
	AHI, per 1-unit increase	1.035	1.002–1.068	0.037
	OSA	3.247	1.373–7.677	0.007

Model 1: Odds ratios for Age, Sex, BMI. Model 2: Odds ratios for Age, Sex, BMI, Multiple stents, Overlapping stents, Total length of stent, Total percentage of time of SaO_2_ <90%. Model 3: Odds ratios for Age, Sex, BMI, LDL-C, Multiple stents, Overlapping stents, Total length of stent, Total percentage of time of SaO_2_ <90%. OSA, obstructive sleep apnea; AHI, apnea-hypopnea index; ISR, in-stent restenosis; BMI, body mass index; LDL-C, low-density lipoprotein cholesterol; OR, odds ratio; CI, confidence interval.

**Fig. 4.**
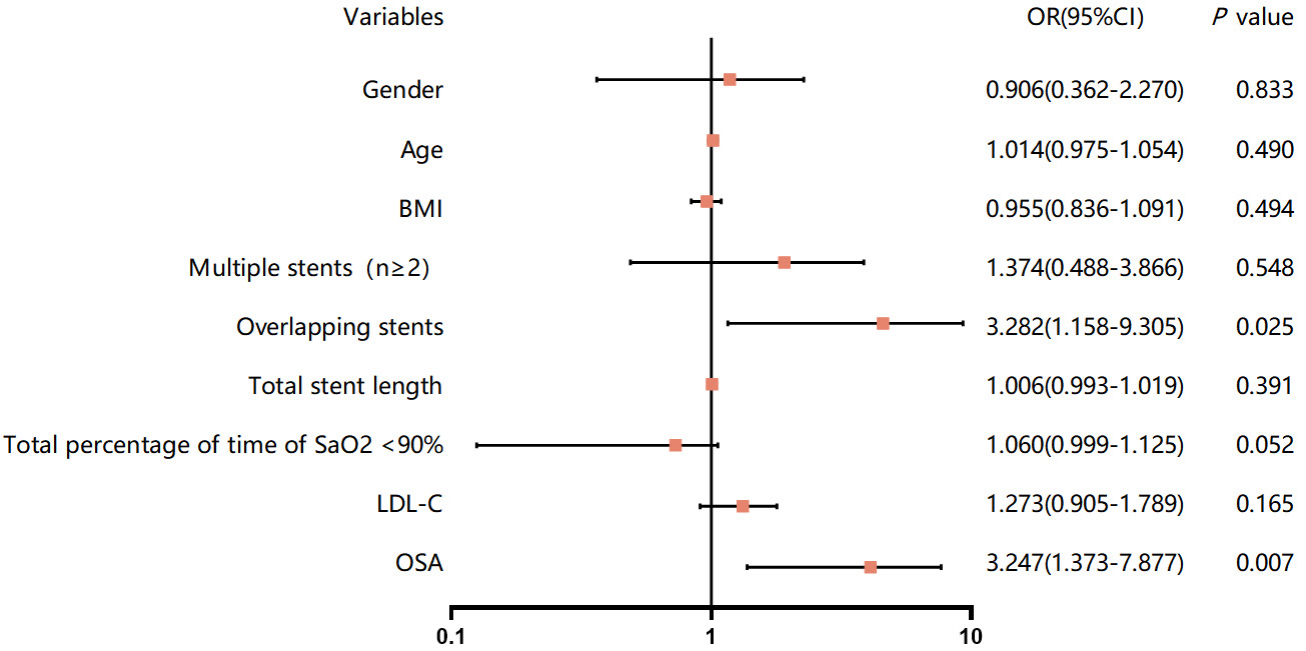
**Forest plot of the multivariable logistic regression analysis model investigating the association between OSA and ISR**. OSA, obstructive sleep apnea; ISR, in-stent restenosis; LDL-C, low density lipoprotein cholesterol; BMI, body mass index; OR, odds ratio; CI, confidence interval.

Second, we converted the AHI into a categorical variable, and the associations between OSA and ISR are shown in Table 3 and Fig. 4. After adjusting for confounders, Model 3 demonstrated a significant and independent correlation between OSA and an increased risk of ISR. The moderate-severe group (AHI ≥15 events/hour) had a threefold increase in the risk of ISR after selective DES implantation (OR: 3.247, 95% CI: 1.373–7.677, *p* =0.007). Moreover, we noticed that the occurrence of overlapping stents constituted an additional independent risk factor for ISR within the context of the fully adjusted model (Model 3). The incidence of ISR was three times greater in patients with stent overlap than in those without (OR: 3.282, 95% CI: 1.158–9.305, *p* = 0.025).

**Fig. 5.**
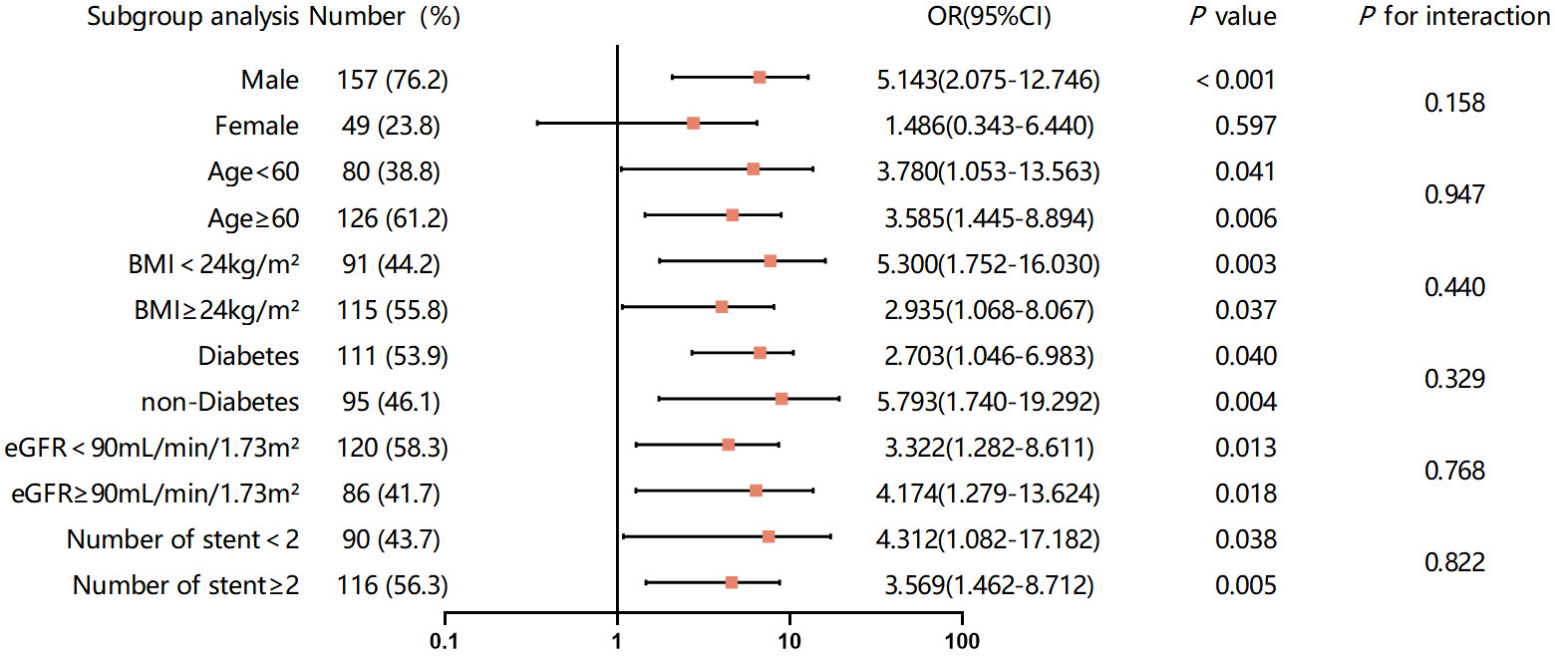
**Forest plot investigating the association between OSA and ISR in subgroup analysis**. OSA, obstructive sleep apnea; ISR, in-stent restenosis; BMI, body mass index; eGFR, estimated glomerular filtration rate; OR, odds ratio; CI, confidence interval.

Subsequently, we also evaluated the association between OSA and ISR in various subgroups (Fig. 5). A correlation between OSA and ISR was primarily observed in males aged ≥60 years with a BMI <24 kg/m^2^, an eGFR <90 mL/min/1.73 m^2^, nondiabetic status, and ≥2 implanted stents after adjusting for baseline variables with *p* < 0.1 in the univariate analysis. Furthermore, this trend persisted within the various subgroups of patients aged <60 years (*p* = 0.041), with a BMI ≥24 kg/m^2^ (*p* = 0.037), with diabetes (*p* = 0.040), with an eGFR ≥90 mL/min/1.73 m^2^ (*p* = 0.018), and with <2 implanted stents (*p* = 0.038). We evaluated the stability of the relationship between OSA and ISR through a subgroup analysis. The results showed that this relationship remained consistent across most subgroups.

## 4. Discussion

In this retrospective analysis, the correlation between OSA and ISR in a subset of patients with CHD who underwent elective implantation of DESs was examined during the season. The critical observations of our research include the following: (1) A statistically significant link exists between the AHI and an elevated incidence of ISR in the setting of DES deployment, establishing the AHI as a standalone predictor. (2) The likelihood of experiencing ISR after drug-eluting stent placement increases with increasing AHI scores. (3) Across both categorical and continuous variables, the presence of OSA is an independent factor affecting ISR risk, as verified by models adjusted comprehensively. (4) The incidence of ISR is increasing among individuals receiving overlapping stents during PCI. At present, there is no report on the relationship between in-stent restenosis and obstructive sleep apnea after DESs. This study provides a new perspective and thinking direction for ISR research. Our study found that AHI has a certain predictive effect on DES-ISR, which provides a basis for early screening and diagnosis of patients with suspected OSA after PCI in clinical practice. At the same time, it also provides future research directions for the management of OSA patients after PCI to a certain extent, such as early OSA treatment, and to a certain extent, AHI has a certain role in predicting DES-ISR. It may reduce the risk of ISR and unnecessary economic burden.

Since its invention in 1979, PCI has become an important treatment method for CHD patients worldwide and has improved the survival rate and quality of life of CHD patients [26]. A series of pathophysiological advances such as plaque prolapse, elastic wall retraction, constrictive remodeling, neointimal hyperplasia, and new atherosclerosis occur in ISR after PCI and seriously affect the prognosis of CHD patients [6]. Compared with bare metal stents, DESs demonstrate a decreased likelihood of restenosis and the necessity for revascularization of the targeted lesion [27]. However, 10.6% of patients still receive PCI due to ISR lesions every year, and the demand for ISR and target lesion revascularization is increasing at an annual rate of 1–2% [3, 4, 7]. Even after successful balloon angioplasty for ISR, 21.4% of patients still underwent target lesion revascularization [8]. Patients who underwent PCI for ISR had a greater risk of MACEs, including myocardial infarction and repeat revascularization, than patients who underwent PCI for the primary lesion [28]. The mechanism of ISR is complex, but it may be related to biological, procedural, anatomical, and stenting factors, and among these biological factors, age, obesity, and female sex have been more commonly reported [3]. A previous study have reported that patients with lower hemoglobin levels have a greater risk of ISR [27]. This finding suggested that the ISR is related to tissue ischemia and hypoxia diseases such as anemia. In our investigation, we observed no notable difference in hemoglobin levels between patients with ISR and those without ISR. However, alongside a higher AHI, a larger proportion of ISR patients experienced less than 90% of their sleep time within the normal SaO_2_ range compared to non-ISR patients.

OSA is a prevalent sleep-related breathing disorder characterized by decreased airflow and apnea episodes. This condition causes intermittent hypoxia, which leads to low blood oxygen, elevated carbon dioxide levels, disrupted sleep patterns, frequent awakenings during the night, increased effort to breathe, and heightened autonomic nervous system activation [29, 30]. The 2021 European Society of Cardiology (ESC) Guidelines for the Clinical Prevention of Cardiovascular Disease indicated an association between sleep disorders or reduced sleep duration and a heightened risk of cardiovascular disease. Among the most widespread sleep-related breathing disorders identified was OSA [31]. Previous reviews have demonstrated that OSA plays a considerable role in the pathophysiology of several cardiovascular diseases [9, 10, 11]. A meta-analysis demonstrated a one-fold increase in coronary revascularization in patients with OSA after successful PCI [32]. Following a median monitoring period of 1.9 years post-PCI among 141 patients, the raw occurrence rate of MACEs, including cardiovascular mortality, nonfatal myocardial infarction, and unplanned coronary revascularization, was observed to be greater among patients with OSA, at 18.9%, compared to 14% in those without OSA [15]. In particular, patients experiencing ACS who also have moderate to severe OSA face a poorer prognosis than those with normal or mild OSA [18]. This was also observed in our study, where patients with moderate-severe OSA had a nearly 20% greater incidence of ISR after elective DES implantation than patients with normal-mild OSA, and individuals within the ISR group exhibited a greater overall AHI in comparison to those in the non-ISR group.

In recent years, many scholars have designed numerous clinical trials to investigate the correlation between OSA and the prognosis of CHD patients and its diagnostic index (AHI). Xiaofan Wu* et al*. [33] demonstrated that patients with untreated moderate-to-severe OSA exhibited a greater need for revascularization than those receiving appropriate treatment for their condition, indicating a significant association between untreated moderate or severe OSA and an elevated risk of revascularization. LM Yao reported a 37% incidence of coronary ISR in patients with OSA during 6 months of follow-up, which was greater than that in patients without OSA [34]. In another study, a follow-up conducted at an average of 7 months involving 78 patients with CHD who were treated with bare-metal stents, revealed that those suffering from OSA experienced a significantly greater rate of late lumen loss, an indicator of restenosis. Stepwise multiple linear regression analysis revealed that an AHI >10/h was a significant predictor of late lumen loss [22]. Research conducted by Lee and colleagues revealed that there were no significant differences in the occurrence of stent-related adverse events, such as target lesion revascularization and in-stent thrombosis, between the groups with and without OSA [15]. However, Yumino D and his team [19] observed an increased incidence of binary restenosis in patients diagnosed with OSA versus those free from OSA at the 6-month post-PCI follow-up of patients with coronary bare-metal stent implantation. Building on these insights, our current investigation identified OSA and the AHI as independent predictors for the development of ISR following the implantation of DESs. This discovery offers a partial explanation for the notable yearly rate of DES-ISR occurrence, even amidst the prevalent adoption of DESs. In a study that included 2717 adults with moderate to severe OSA and coronary or cerebrovascular disease, the primary outcome measure was the incidence of myocardial infarction, cardiovascular death, stroke, or hospitalization for unstable angina, heart failure, or transient ischemic attack. The addition of CPAP therapy to standard care did not reduce cardiovascular events, suggesting a complex relationship between OSA treatment and cardiovascular outcomes [35], which might imply that OSA is not directly associated with cardiovascular events. Nevertheless, it is notable that this study exclusively involved patients who were already diagnosed with cardiovascular and cerebrovascular diseases and omitted those who developed CHD after stent implantation. As a result, to thoroughly evaluate whether interventions targeting the treatment of OSA could beneficially influence the prevention of DES-ISR, future multicenter, prospective, and randomized studies are needed.

The exact mechanism underlying the close association between OSA and DES-ISR remains unclear. ISR is mainly caused by endothelial and vascular damage caused by stent implantation, which stimulates inflammation and fibroblast proliferation, leading to vascular endothelial hyperplasia, excessive neointimal hyperplasia and neoatherosclerosis, eventually causing ISR [3]. OSA is characterized by hypoxemia, autonomic nerve dysfunction, arousal, intrathoracic pressure changes, and hypercapnia, leading to adverse pathophysiological responses, including inflammation, atherosclerosis, endothelial dysfunction, sympathetic activation, and hypercoagulable states [10]. Hypoxia may be one of the possible mechanisms by which OSA leads to ISR. Hypoxia can lead to vascular endothelial cell proliferation and neovascularization [36], both of which are necessary conditions for the occurrence and development of DES-ISR [3, 37]. Compensatory responses to hypoxia contribute to the development of ISR, including a hyperdynamic state characterized by increased cardiac output, left ventricular hypertrophy, progressive cardiac enlargement, and proatherogenic effects. These physiological adjustments, while initially serving to offset reduced oxygen levels, can ultimately exacerbate the risk of ISR by promoting adverse cardiovascular changes [38]. In addition, the mechanism of ISR development may also be related to many pathophysiological processes in OSA that promote inflammation and oxidative stress, which directly leads to vascular endothelial dysfunction (a pro-atherogenic factor) while reducing nitric oxide utilization and endothelial repair capacity [39, 40]. Other possible explanations for endothelial dysfunction in OSA patients include apoptosis, interactions between circulating inflammatory cells and endothelial cells, damage repair processes, and microparticles [41]. However, to further elucidate the potential mechanisms by which OSA contributes to the development of ISR, additional experimental data are essential. Such research would provide valuable insights into the underlying pathological processes, potentially leading to more effective strategies for preventing ISR in patients with OSA.

Several limitations in our present study should be noted. First, this is a single-center, retrospective, observational study. As a result, we were not able to determine the causality between OSA and ISR. Hence, future randomized clinical trials (RCTS) are required to examine whether actively treating OSA could be beneficial in the prevention of ISR. Second, selection bias may have affected the results of this study. Our study only included CHD patients who underwent elective drug-eluting stenting and were readmitted due to symptoms of CHD ranging from 12 to 26 months, which maylimit the generalizability of our findings to patients with ACS. Third, in this study our ISR was determined by visual judgment under contrast by two sophisticated interventional cardiologists, rather than more accurate intravascular imaging such as optical coherence tomography. Fourth, we only monitored the patients’ sleep during one night of hospitalization, which may not reflect the patients’ long-term sleep. Finally, although we adjusted for as many factors that might affect ISR as possible in the multivariable logistic regression analyses, there were still potential confounders, that could not be completely eliminated. For example, in the final adjusted model, only LDL-C, the most important risk factor of CHD, was included except for the variables with *p* value < 0.1 in the univariate analysis. Other potential confounding factors, such as maximal expansion pressure, may not be meaningful in the univariate analysis, but may produce meaningful results in the multivariate analysis. Additionally, although we included glycated hemoglobin, we did not include blood glucose. At the same time, because most of the patients included in the analysis did not have inflammatory markers detected, we did not include them in the baseline data, which may cause a certain bias in the study results.

Therefore, we recommend that future prospective multicenter randomized controlled trials: (1) Patients should be monitored for long-term sleep to overcome the limitation of long-term fluctuations in sleep pattern and quality that cannot be reflected by single sleep monitoring; (2) More accurate intravascular imaging techniques such as optical coherence tomography (OCT) should be used to determine the presence and extent of ISR to improve the accuracy of the study; (3) Studies with a larger sample size and more comprehensive multivariate adjustment may be conducted to exclude all potential confounders and further investigate the cost-effectiveness ratio of CPAP treatment for OSA in preventing ISR and the role of this preventive strategy in reducing the health economic burden; (4) The independent effects of sleep parameters other than AHI, such as sleep depth and sleep architecture, on the risk of DES-ISR should be further investigated in order to more fully understand the mechanism of OSA on the risk of DES-ISR.

## 5. Conclusions

In patients undergoing elective drug-eluting stenting, OSA, characterized by interrupted respiration during sleep, is an independent risk factor for ISR. Compared to individuals with normal-to-mild OSA, individuals with moderate-to-severe OSA have a threefold increased risk of ISR after PCI. Moreover, as a diagnostic parameter for OSA, an evaluation in the AHI is correlated with an increased risk of ISR. The greater the AHI is, the more likely the patient is to have ISR after DES implantation.

## Data Availability

The datasets used and/or analyzed during the current study are available from the corresponding author on reasonable request.

## Abbreviations

OSA, obstructive sleep apnea; ISR, in-stent restenosis; DES, drug-eluting stent; CHD, coronary heart disease; PCI, percutaneous coronary intervention; AHI, apnea-hypopnea index; MACEs, major adverse cardiac events; ACS, acute coronary syndrome; BMI, body mass index; CPAP, continuous positive airway pressure; TC, total cholesterol; TG, triglyceride; HDL-C, high-density lipoprotein cholesterol; LDL-C, low-density lipoprotein cholesterol; LP (a), lipoprotein a; eGFR, estimated glomerular filtration rate; UA, uric acid; HbA1c, hemoglobin A1c; LVEF, left ventricular ejection fraction; ACEI/ARB, angiotensin-converting enzyme inhibitors/angiotensin receptor blocker; LM, left main artery; LAD, left anterior descending artery; LCX, left circumflex artery; RCA, right coronary artery; OR, odds ratio; CI, confidence interval; ROC, receiver operating characteristic; AUC, area under the curve.

## Supplementary Material

Supplementary material associated with this article can be found, in the online version, at https://doi.org/10.31083/RCM25814.
