# Supplementary material for: Impact of Obstructive Sleep Apnea on In-Stent Restenosis in Coronary Heart Disease Patients after Elective Drug-Eluting Stenting
Source: Rev Cardiovasc Med. 2025 Jan 15;26(1):25814. doi: 10.31083/RCM25814 (PMC11759976; doi:10.31083/RCM25814)
Supplement: Supplementary file 1 [file 2153-8174-26-1-25814-s1.docx]

**Supplementary material**

| **Table 1** Characteristics of patients with non-ISR and ISR | | | |  |
| --- | --- | --- | --- | --- |
| Variables | non-ISR  (n=164) | ISR  (n=42) | *P* value | |
| Age, year | 61.73±10.19 | 62.12±10.60 | 0.436 | |
| Male, n (%) | 40 (24.4) | 9 (21.4) | 0.688 | |
| BMI, kg/m^2^ | 24.48±3.22 | 24.97±2.91 | 0.436 | |
| Obesity, n (%) | 16 (9.8) | 5 (11.9) | 0.681 | |
| Hypertension, n (%) | 105 (64.0) | 25 (59.5) | 0.590 | |
| Diabetes mellitus, n (%) | 87 (53.0) | 24 (57.1) | 0.635 | |
| Current smoking, n (%) | 46 (28.0) | 16 (38.1) | 0.205 | |
| TC, mmol/L | 4.27 [3.47,5.37] | 4.74 [3.59,5.85] | 0.155 | |
| TG, mmol/L | 1.54 [1.11,2.35] | 1.49 [1.17,2.20] | 0.946 | |
| HDL-C, mmol/L | 0.94±0.24 | 0.95±0.20 | 0.785 | |
| LDL-C, mmol/L | 2.81±1.09 | 3.14±1.09 | 0.089 | |
| eGFR, mL/min/1.73m^2^ | 88.0 [72.8,95.6] | 83.9 [69.1,92.5] | 0.389 | |
| UA, umol/L | 395.5±117.9 | 403.1±99.7 | 0.718 | |
| HbA1c | 6.1 [5.6,6.8] | 6.0 [5.5,7.7] | 0.591 | |
| Hemoglobin, g/L | 135 [126,146] | 138 [131,146] | 0.314 | |
| Medications at discharge |  |  |  | |
| Aspirin, n (%) | 162 (98.8) | 41 (97.6) | 1.000 | |
| Ticagrelor, n (%) | 55 (33.5) | 13 (31.0) | 0.751 | |
| Clopidogrel, n (%) | 108 (65.9) | 29 (69.0) | 0.696 | |
| Statin, n (%) | 163 (99.4) | 41 (97.6) | 0.871 | |
| β-block, n (%) | 100 (61.0) | 27 (64.3) | 0.694 | |
| ACEI/ARB, n (%) | 75 (45.7) | 24 (57.1) | 0.187 | |
| Angiography |  |  |  | |
| Multivessel disease, n (%) | 126 (76.8) | 33 (78.6) | 0.810 | |
| LM disease, n (%) | 17 (10.4) | 3 (7.1) | 0.529 | |
| Intervention vessel |  |  |  | |
| LM, n (%) | 5 (3.0) | 3 (7.1) | 0.437 | |
| LAD, n (%) | 93 (56.7) | 25 (59.5) | 0.742 | |
| LCX, n (%) | 54 (32.9) | 12 (28.6) | 0.589 | |
| RCA, n (%) | 61 (37.2) | 21 (50.0) | 0.130 | |
| Type of DES |  |  |  | |
| Sirolimus stent, n (%) | 121 (73.8) | 36 (85.7) | 0.105 | |
| Everolimus stent, n (%) | 44 (26.8) | 6 (14.3) | 0.091 | |
| Multiple stents (n≥2), n(%) | 86 (52.4) | 30 (71.4) | 0.027  0.875 | |
| Minimal stent diameter, mm | 2.75 [2.50,3.00] | 2.75 [2.50,3.00] |  |  |
| Stent overlapping, n (%) | 11 (6.7) | 11 (26.2) | ＜0.001 | |
| Maximal expansion pressure | 16 [16,18] | 18 [16,18] | 0.021 | |
| Total stent length, mm/patients | 39 [28,65] | 58 [36,78] | 0.019 | |
| AHI, events/h | 11.09 [5.74,20.40] | 20.22[11.61,27.50] | **0.001** | |
| Minimal SaO_2_ (%) | 85 [83,89] | 84 [80,88] | 0.107 | |
| Mean SaO_2_ (%) | 94 [93,95] | 95 [92,96] | 0.609 | |
| Total percentage of time of SaO_2_ <90% | 0.41 [0.01,2.22] | 0.69 [0.09,7.17] | **0.017** | |

The data are reported as n (%) for categorical variables and as the mean±SD or median [IQR] for continuous variables. BMI: body mass index; TC: total cholesterol; TG: triglyceride; HDL-C: high-density lipoprotein cholesterol; LDL-C: low-density lipoprotein cholesterol; eGFR: estimated glomerular filtration rate; UA: uric acid; HbA1c: hemoglobin A1c; ACEI/ARB: angiotensin-converting enzyme inhibitors/angiotensin receptor blocker; LM: left main artery; LAD: left anterior descending artery; LCX: left circumflex artery; RCA: right coronary artery; ISR: in-stent restenosis; AHI: apnea-hypopnea index.

| **Table 2** Association of OSA and ISR in univariate logistic regression analysis | | | |  |
| --- | --- | --- | --- | --- |
| Variables | OR | 95%CI | *P* value | |
| LDL-C | 1.293 | 0.959-1.743 | 0.092 | |
| Everolimus stent | 0.455 | 0.179-1.153 | 0.097 | |
| Multiple stents (n≥2) | 86 (52.4) | 30 (71.4) | ＜0.001 | |
| Stent overlapping | 4.935 | 1.966-12.392 | 0.001 | |
| Maximal expansion pressure | 1.132 | 0.992-1.290 | 0.065 | |
| Total stent length | 1.011 | 1.002-1.020 | 0.021 | |
| OSA | 3.712 | 1.774-7.770 | 0.001 | |
| AHI | 1.044 | 1.017-1.071 | 0.001 | |
| Minimal SaO_2_ | 0.958 | 0.914-1.003 | 0.068 | |
| Total percentage of time of SaO_2_ <90% | 1.084 | 1.021-1.150 | 0.008 | |

LDL-C: low-density lipoprotein cholesterol; OSA: obstructive sleep apnea; AHI: apnea-hypopnea index.


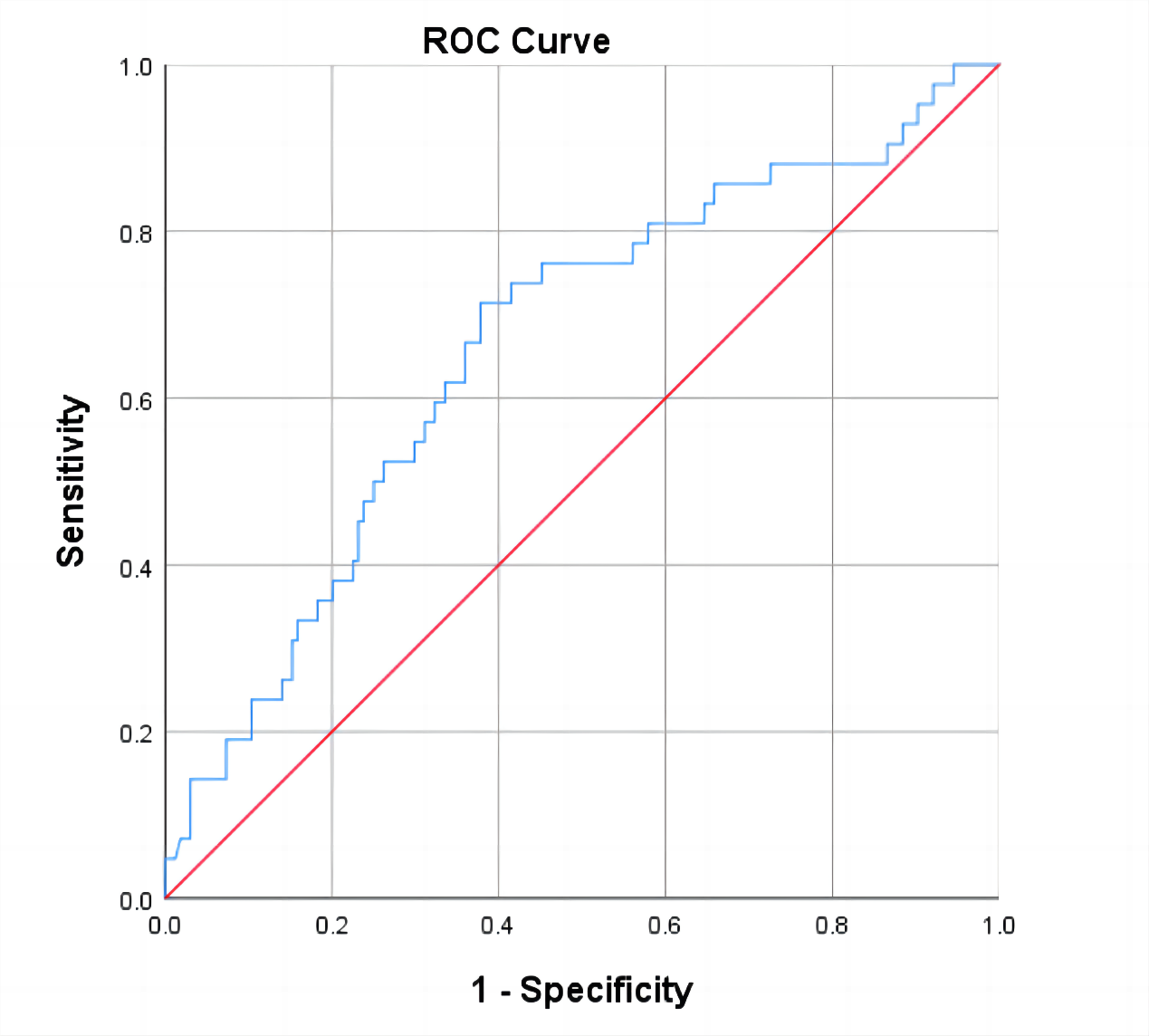


Figure 1 Receiver operating characteristic curve analysis of the AHI to predict DES-ISR
